# Meeting present and future challenges in sustainable horticulture using virtual plants

**DOI:** 10.3389/fpls.2013.00443

**Published:** 2013-11-05

**Authors:** Gerhard Buck-Sorlin, Mickaël Delaire

**Affiliations:** Agro Campus OUEST Centre d'Angers, Institut de Recherche en Horticulture et Semences, Institut National d'Horticulture et de Paysage, UMR 1345Angers, France

**Keywords:** apple, FSPM, typology, classification, horticulture, models, biological

## The purpose of models and the modeling exercise

In order to better understand the significance of models in practical horticulture we have to put ourselves into the position of the horticulturist (orchard grower, consultant …) and appreciate first of all some skepticism toward modeling. This does not necessarily mean a downright refusal to use models: on the contrary, it is often the result of an initial keen interest coupled with high expectations in modeling as such, frequently followed by disillusionment that the high hopes were not satisfied; that *the* model as a response to very specific problems and questions does not exist; that the models offered were remote from practice, unreliable, or too complicated to use. The special case of orchards as perennial production systems, poses a further challenge, for two reasons. Firstly, a rapidly increasing complexity: if considered it renders the model difficult to parameterize, in-transparent and expensive; if, on the other hand, it is neglected it is fair to question the model's added value compared to the extensive experience of a grower. Secondly, a perennial production system represents the integral of a unique combination of location and history of production, and the interaction between the genotype with the local environment, making a generalization more difficult than for annual crops. So, what should be done about it? First of all, models are tools, equivalent to other experimental tools, not ultimate purposes: it is up to the researcher to get this message across to the user, to remove any misunderstandings regarding models as “perfect solutions” delivering “absolute answers,” and to lower exaggerated hopes. Next, a common language between the researcher and the practitioner must be found, because in practice wires get crossed, not only with respect to the technicalities of the modeling approach but also with respect to the goals of the modeling exercise. Despite that, modelers and people from practice do actually pursue the same goal: trying to integrate the complex information (measured and observed) about a given system, in order to obtain a result (admittedly, opinions as to what is an acceptable result diverge, ranging from a decision-support tool to a publication…).

## The “true” challenges of modeling

**Figure 1:**
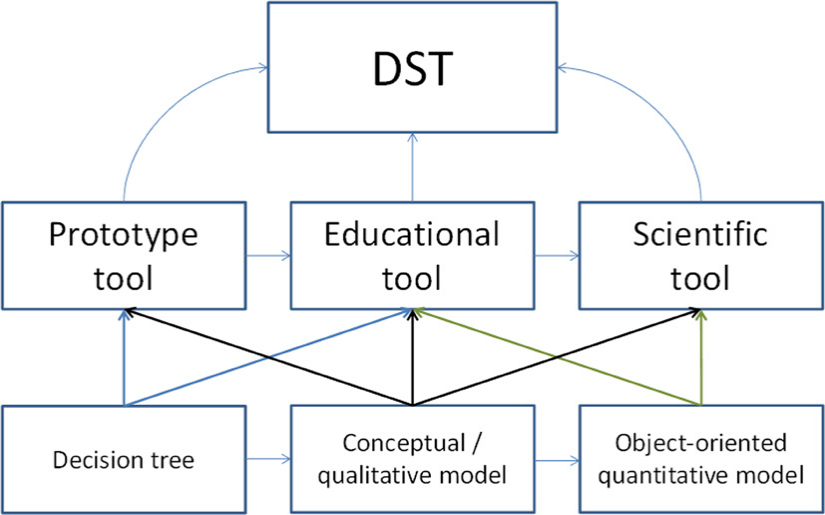
**Typology of models used in horticulture.** DST, Decision Support Tool. For explanations see text.

In order to advance modeling as a scientific technique and to render its products more accessible to practice—in other words to come down the ivory tower of theoretical biology and applied mathematics in which many biological models and their authors reside—it is important to communicate the true challenges of modeling (which go beyond the predictions coming out of the black box of the model). The three principal challenges are:

- To design models as tools of information exchange between the different types of experts and practitioners of a horticultural sector (e.g., orcharding). Using the formalism developed to construct the model it must be possible to confront the different implicit representations of the stakeholders and to further let these representations evolve using a co-construction approach (similar to developing the Wikipedia), considering at the same time representations added from the “outside” (by scientists) and those belonging to the stakeholders working in practice. In this respect it is crucial that the modelers provide communication tools that permit to state explicitly and simply the conceptual bases of the formalism used to construct the model. This type of models is usually referred to as decision-support tools (DST), but actually DST is a too general and too advanced term for them as DST can also be derived from scientific and pedagogic models (Figure 1). We will instead call them prototype tools as they allow us to integrate and structure different knowledge types about a particular system into a simplified representation that serves as a first step toward developing either true DSTs or scientific tools. A prototype tool, like the prototype of a new car, can thus be conceived as an early version of the model, in which the boundaries, elements, behavior and levels of the system are already roughly specified.
- To design models as systems analysis tools which permit to capture emergent phenomena and predetermined breaking points. The computational capacity, available thanks to progress in hardware technology allows testing an infinite diversity of scenarios and combinations of potential actions. This type of models could be termed scientific tools.
- To design educational models which convey textbook knowledge in a visual and dynamic form in order to serve as teaching material in university courses, e-learning, or as instructions for orchard workers (e.g., technique and consequences of pruning and shoot bending). We will term this type educational tool.

All of the three types can be developed into DSTs (Figure 1), depending on the level of prior knowledge of the user, the design of the model and the modeling paradigm used (e.g., a decision tree, a conceptual model, or an object-oriented model), and the kind of decision-support envisaged (e.g., initial orchard design, prediction of fruit quality, scheduling of pest control measures as part of integrated biological production…).

An additional challenge for biological modeling is the change of scale, i.e., the integration from a lower to a higher scale, and the differentiation from a higher to a lower scale. Integration of knowledge from the gene to the field, or even to the production region or the natural ecological zone is fanciful among plant scientists and this despite the lack of a clear methodology. From the shelter of their disciplines (genetics, physiology, ecophysiology, agronomy, ecology), scientists hypothesize that modeling might be the best choice to try to simplify the complexity encountered at each level and at the transgression from one level to the next. Accordingly, models have been developed to document the knowledge of a certain discipline and at a certain hierarchical scale. However, the concepts and tools to scale up and down, let alone to integrate “horizontally,” i.e., to meaningfully link two or more disciplines (beyond the use of a common database…), are still in their infancy.

## What type of modeling?

Different approaches to crop modeling have been developed in the past (Vos et al., 2010). The most recent approach among them, functional-structural plant modeling or “virtual plants” currently seems the most promising, especially in horticulture which distinguishes itself from agriculture by its enormous genetic and structural diversity (cultures and production methods) and a high technological investment (see greenhouse horticulture). FSPM refers to a paradigm for the description of a plant by creating a computer model of its structure and selected physiological and physical processes, at different hierarchical levels: organ, plant individual, canopy, and in which the processes are modulated by the local environment (Buck-Sorlin, 2013). Within the modeling paradigm of FSPM there exist types and gradations along the following opposite pairs: descriptive versus explanatory, stochastic versus deterministic, or dynamic versus static. From a programming paradigm point of view the most meaningful criterion is the one which distinguishes procedural, object-oriented, and rule-based approaches. Independent of the paradigm chosen in the end, all approaches have in common that they serve to represent a complex reality by decomposing, modularizing and simplifying it into (arguably) atomic units, in order to make it easier to handle, to understand, and to anticipate changes. The method of decomposition necessarily is often biased and depending on the eye and the intention of the observer, thence the importance of fixing the objectives of the model ahead of the actual modeling exercise. On the other hand, a deliberate effort can be made to approach the description of the system in an “objective” way, i.e., trying to be independent of any intention or subjective bias (knowing fully well, of course, that this is only possible as an approximation since all system descriptions are ultimately subjective). It is in this respect useful to make a distinction between procedural and object-oriented modeling: In procedural modeling (equivalent to classical crop modeling: applied in horticulture and agriculture since more than forty years) the available information is structured according to a chain of more or less concrete processes; ultimate aims of the model are fixed beforehand and the process of knowledge acquisition (and subsequent use in the model) is deductive and top-down. Contrary to this, in object-oriented modeling the available information is structured around objects (their traits and relations to each other) that have been identified a priori as relevant and/or characteristic, and in which knowledge acquisition is inductive and bottom-up.

## The notion of structure in FSPM

Though perfectly clear to everyone involved in research on FSPM, the idea of structure in Functional-Structural Plant Models regularly leads to misunderstandings outside the community. *Structure* in an FSPM, especially with respect to woody plants, corresponds to plant architecture, more precisely at the organ and plant individual level, not more and not less. This notion of architecture comprises the topology and the geometry of plant organs in relative and absolute coordinates and also helps to improve the definition of the interfaces with the microenvironment, which latter is both surrounding and being modified by the architecture of the plant (part) located inside it. The precise description of organ location and orientation by topology and geometry is the basis for quantitatively modeling the transport of carbon, water and minerals between sources and sinks. Many FSPM exist that have implemented the process chain from light interception to photosynthesis, assimilate distribution and growth in terms of organs extension or increase in biomass. On the other hand, FSPMs which also consider the feedback of a change in architecture (organogenesis by bud break) on, e.g., photosynthesis rate are rare.

## A multi-scale object-oriented FSPM of the apple production system

Heterogeneity, within the tree and in different years, in apple (*Malus* × *domestica*) fruit production poses a number of challenges: fruit quality and number (fruit load) can vary as a function of genotype, climate, or an interaction between these factors. Previous experimental work conducted by the ecophysiology team at Angers has shown that the carrying branch or limb is an apt experimental system for the investigation of these phenomena if certain key variables at the next lower (organ) and next higher (plant) scale are considered at the same time.

We have recently started to create a prototype model of the apple limb, in order to improve our knowledge about the role of plant architecture for the formation of fruit quality. This model is object-oriented and covers three hierarchical, consecutive scales: organ, branch, and plant; a number of physiological processes is defined for each organ (e.g., photosynthesis, growth, respiration…); transport of sugar, water and minerals will be defined at the organ level using rules that apply rate equations to pairs of topologically joined organs (leaf–internode, internode–internode, internode–petiole, petiole–fruit), whilst the equations will be integrated in parallel with an embedded ODE solver (Hemmerling, 2012). At the level of the organ type “fruit” a modification of the “Virtual Fruit” model (Génard et al., 2007) will be employed. Since we are interested in the production (quantity and quality) of a given year, without having to reconstruct the history of the tree at each simulation run, we devised an initiation rule which at the start of each run puts in place the initial (measured) plant architecture as encountered in spring before bud break, of that year.

## Outlook

The modeling exercise described here is exemplary for many modeling projects in horticulture, independently of whether these are departing from existing modules or from scratch. In the first place a conceptual model is elaborated and then qualitatively validated. Existing modules that have been parameterized for a certain fruit (e.g., peach) eventually need to be reparameterized, as in the Virtual Fruit model, where also a partial rewriting of the equations is necessary to make it applicable for apple. After proper calibration of modules (this can be done by doing sensitivity analyses under standard conditions), the latter can be tentatively combined, recalibrated and eventually validated using an external data set, with the ultimate aim to obtaining a model which can predict fruit quality as a function of the genotype and a specific production environment.
